# In vaginal fluid, bacteria associated with bacterial vaginosis can be suppressed with lactic acid but not hydrogen peroxide

**DOI:** 10.1186/1471-2334-11-200

**Published:** 2011-07-19

**Authors:** Deirdre E O'Hanlon, Thomas R Moench, Richard A Cone

**Affiliations:** 1 Mucosal Protection Laboratory, Thomas C Jenkins Department of Biophysics, Johns Hopkins University, 3400 North Charles Street, Baltimore MD 21218, USA; 2 ReProtect Inc., 703 Stags Head Road, Baltimore MD 21704, USA

## Abstract

**Background:**

Hydrogen peroxide (H_2_O_2_) produced by vaginal lactobacilli is generally believed to protect against bacteria associated with bacterial vaginosis (BV), and strains of lactobacilli that can produce H_2_O_2 _are being developed as vaginal probiotics. However, evidence that led to this belief was based in part on non-physiological conditions, antioxidant-free aerobic conditions selected to maximize both production and microbicidal activity of H_2_O_2_. Here we used conditions more like those *in vivo *to compare the effects of physiologically plausible concentrations of H_2_O_2 _and lactic acid on a broad range of BV-associated bacteria and vaginal lactobacilli.

**Methods:**

Anaerobic cultures of seventeen species of BV-associated bacteria and four species of vaginal lactobacilli were exposed to H_2_O_2_, lactic acid, or acetic acid at pH 7.0 and pH 4.5. After two hours, the remaining viable bacteria were enumerated by growth on agar media plates. The effect of vaginal fluid (VF) on the microbicidal activities of H_2_O_2 _and lactic acid was also measured.

**Results:**

Physiological concentrations of H_2_O_2 _(< 100 μM) failed to inactivate any of the BV-associated bacteria tested, even in the presence of human myeloperoxidase (MPO) that increases the microbicidal activity of H_2_O_2_. At 10 mM, H_2_O_2 _inactivated all four species of vaginal lactobacilli but only one of seventeen species of BV-associated bacteria. Moreover, the addition of just 1% vaginal fluid (VF) blocked the microbicidal activity of 1 M H_2_O_2_. In contrast, lactic acid at physiological concentrations (55-111 mM) and pH (4.5) inactivated all the BV-associated bacteria tested, and had no detectable effect on the vaginal lactobacilli. Also, the addition of 10% VF did not block the microbicidal activity of lactic acid.

**Conclusions:**

Under optimal, anaerobic growth conditions, physiological concentrations of lactic acid inactivated BV-associated bacteria without affecting vaginal lactobacilli, whereas physiological concentrations of H_2_O_2 _produced no detectable inactivation of either BV-associated bacteria or vaginal lactobacilli. Moreover, at very high concentrations, H_2_O_2 _was more toxic to vaginal lactobacilli than to BV-associated bacteria. On the basis of these *in vitro *observations, we conclude that lactic acid, not H_2_O_2_, is likely to suppress BV-associated bacteria *in vivo*.

## Background

Bacterial vaginosis (BV) is a common, frequently recurrent condition in which a relatively sparse, lactobacilli-dominated vaginal microbial community is replaced by a dense mixture of Gram-variable and Gram-negative bacteria. Since hydrogen peroxide (H_2_O_2_) is a broad-spectrum microbicidal disinfectant, the ability of some strains of lactobacilli to produce H_2_O_2 _suggested that these strains might help prevent BV. Women with H_2_O_2_-producing lactobacilli are less likely to have BV than are women without H_2_O_2_-producing lactobacilli [1-3]. Additionally, H_2_O_2_-producing lactobacilli were shown to inactivate several species of BV-associated bacteria under aerobic *in vitro *conditions and in the absence of the anti-oxidants present in physiological fluids [4,5]. Lactobacilli strains that produce H_2_O_2 _are now being selected for developing vaginal probiotics [6-8].

However, recent work in our laboratory [9] has shown that under the hypoxic conditions that generally prevail in the vagina, H_2_O_2 _production by vaginal lactobacilli is undetectable (detection threshold 10 nM). Even with extended aerobic exposures *in vitro*, the mean H_2_O_2 _concentration achieved by lactobacilli in vaginal fluid (VF) was only 23 μM ± 5 μM, approximately 100-fold lower than the concentration of H_2_O_2 _achieved by lactobacilli under aerobic *in vitro *conditions in the absence of anti-oxidants. Furthermore, VF has sufficient anti-oxidant activity to block the microbicidal activity of H_2_O_2 _even when H_2_O_2 _is supplied at concentrations much higher than lactobacilli are capable of producing. We believe these findings make protection by H_2_O_2 _implausible *in vivo*.

Vaginal lactobacilli produce several target-specific antimicrobial factors, including bacteriocins [10,11], bacteriocins-like substances [12], and selective ligands [13]. However, given the broad spectrum of BV-associated bacteria and the diverse reproductive tract infections that occur more frequently in women with BV, we chose to compare the microbicidal activities of the most robust broad-spectrum antimicrobials that lactobacilli are known to produce: H_2_O_2 _and lactic acid. Hydrogen peroxide causes oxidative stress in bacterial cells [14], at least partially by oxidizing sulphydrals, and by oxidizing free iron to produce hydroxyl radicals that react with nucleic acids [15]. Lactic acid, under acidic conditions, can permeate cell membranes, acidify the cytosol [16,17], and induce osmotic stress [18]. Lactic acid has also been shown to have broad spectrum activity against Gram-negative bacteria, probably by weakening the cell wall [19]. To clarify whether cytosolic acidification is the primary anti-microbial action of lactic acid, we also observed the effects of acetic acid, which is elevated during episodes of BV [20,21], and which, by being smaller and more lipid soluble, can acidify cytosol more rapidly than lactic acid [22].

The aim of this study, therefore, was to compare the antimicrobial actions of H_2_O_2_, lactic acid, and acetic acid on BV-associated bacteria *and *on vaginal lactobacilli under anaerobic growing conditions that approximate the hypoxic environment of the vagina [23]. We also examined the effects of VF, which consists of endocervical mucus that has entered the vagina and mixed with shed cells and transudated fluid from the vaginal epithelium. VF is acidified with lactic acid to ≤ pH 4.5 if the vaginal microbial community is dominated by lactobacilli [24]. We selected seventeen different species of bacteria that have been associated with BV by either bacteriological or molecular methods. We also studied four of the most common species of vaginal lactobacilli, including the recently identified *Lactobacillus iners*. We tested microbicidal activity at pH 4.5 (the highest non-menstrual vaginal pH expected in the absence of BV) and pH 7.0 (the approximate pH of the vagina during menses, and briefly following exposure to semen.

## Methods

All materials and reagents were supplied by Sigma-Aldrich Inc., (St. Louis MO), unless otherwise specified; all microorganisms were supplied by the American Type Culture Collection (Manassas VA).

### Lactobacilli

*Lactobacillus crispatus *ATCC 33820 was grown in ATCC medium 1490 (Modified chopped meat medium), *L. jensenii *ATCC 25258 and *L. gasseri *ATCC were grown in ATCC medium 416 (Lactobacilli MRS broth), *L. iners *ATCC 55195 was grown in ATCC medium 1685 (NYC III medium). Each species was grown anaerobically without agitation at 37°C for 24 hours before use in an experiment.

### Bacteria associated with bacterial vaginosis

*Gardnerella vaginalis *ATCC 14018 was grown in ATCC medium 1685 (NYC III medium). *Prevotella bivia *ATCC 29303, *Prevotella corporis *ATCC 33547, *Anaerococcus prevotii *ATCC 14952, *Fusobacterium nucleatum *ATCC 25586 and *Porphyromonas levii *ATCC 29147 were all grown in ATCC medium 1490 (Modified chopped meat medium); *Bacteroides ureolyticus *ATCC 33387 was grown in ATCC medium 1490 with formate and fumarate. *Peptostreptococcus anaerobius *ATCC 27337, *Anaerococcus tetradius *ATCC 35098, *Atopobium vaginae *ATCC BAA-55, *Megasphaera elsdenii *ATCC 25940, and *Propionibacterium acnes *ATCC 6919 were all grown in ATCC medium 1053 (Reinforced Clostridial medium) supplemented with 5% defibrinated rabbit blood (Colorado Serum Company, Denver CO). *Ureaplasma urealyticum *ATCC 27618 was grown in ATCC medium 1331 (Urea broth); *Mobiluncus curtisii *ATCC 35241 and *Mobiluncus mulieris *ATCC 35239 were grown in BBL™ Schaedler medium (Becton, Dickinson and Company, Sparks MD). *Mycoplasma hominis *ATCC 23114 was grown in ATCC medium 243 (Mycoplasma medium). *Micromonas micros *ATCC 33270 was grown in ATCC medium 1102 (Chopped meat medium) supplemented with 0.1% each of cellobiose, maltose, starch, and Tween 80. Each species was grown anaerobically in a 50 mL volume of its recommended growth medium without agitation at 37°C for 24 or 48 hours before use in an experiment, yielding bacterial concentrations between approximately 10^6 ^and 10^9 ^colony-forming units (cfu) per mL (48 hour incubations were used for bacteria that failed to produce consistently = 10^6 ^cfu/mL after 24 hour incubations). The relatively high concentrations of bacteria used were chosen both to increase the dynamic range of the experiments (i.e., large numbers of bacteria permit a more meaningful quantification of observed inactivation), and to reflect the high density of bacteria seen *in vivo *[25].

### Microbicidal activity

Experimental media for each organism were prepared by adding H_2_O_2_, lactic acid, or acetic acid to the appropriate growth medium for that organism; they were not added to control media. For experiments using H_2_O_2_, both experimental and control media contained 50 mU/mL human myeloperoxidase (MPO). All growth medium formulations contained at least ten-times more than the 1 mM concentration of chloride ions required for full activity of a myeloperoxidase-halide-H_2_O_2 _microbicidal system [26]. Aliquots of each experimental and control medium were titrated with sodium hydroxide or hydrochloric acid as necessary to obtain a pH of either 4.5 or 7.0 (with allowance made for the change in pH that would occur when an aliquot of bacterial culture was added, as described below).

Bacterial cultures were gently agitated immediately before use. A 100 μL aliquot of culture was added to 9.9 mL of each control or experimental medium; media and bacteria were then incubated anaerobically at 37°C. Two replicate samples were removed from control and experimental conditions after ten minutes, thirty minutes, one hour, and two hours exposure. Each sample was then serially diluted with the appropriate growth medium containing 200 mM HEPES (pH 6.8-7.2 depending on growth medium) and track-plated [27] onto the appropriate growth medium containing 1.5% (w/v) ultrapure agar (USB Corporation, Cleveland OH). The pH of each experimental or control medium was re-measured after the experiment to confirm it had remained within 0.1 pH units of the starting pH. Agar plates were incubated anaerobically at 37°C for 24 or 48 hours, until colonies could be easily distinguished and counted. Colonies on some plates were recounted after a further 48 hours incubation to allow for extended lag-phases in treated cells; however, no further changes in colony-counts were observed. Each experiment was independently repeated at least four times.

### Bacterially-depleted vaginal fluid

#### Participants

The study was carried out at the Johns Hopkins University Homewood campus. Each participant gave written informed consent under a protocol approved by the Homewood Institutional Review Board on the Use of Human Subjects at Johns Hopkins University. Participants were required to be between 18 and 45 years old, in good general health, at least three days past the most recent menstruation or unprotected penile-vaginal intercourse, at least three weeks past the most recent use of vaginal or systemic antimicrobials, and free from vaginal symptoms (discharge, odor, itching, or pain). Results from twenty-two samples donated by eight participants are reported here; the group comprised roughly equal numbers of non-Hispanic whites, blacks, and Asians, aged between 21 and 44 years old (mean age 27 ± 4 years).

#### Collection of vaginal fluid samples

For these experiments, undiluted non-menstrual VF was collected at the laboratory using the non-absorbent disposable Instead^® ^Softcup™ menstrual device (Evofem Inc., San Diego CA) [28]. The Softcup was vaginally inserted, removed, and placed in a conical centrifuge tube. The collected VF was removed from the Softcup by centrifugation for one minute at 500 *g*; the Softcup was then discarded.

A sterile cotton swab was dipped into the collected fluid, rolled out onto a glass microscope slide, and air-dried for later Gram-staining and Nugent-scoring. A total of eight participants donated VF; all samples had Nugent score ≤ 3 and no evidence of leukorrhea (the mean PMNL/hpf of the samples was 2.3). As reported earlier, VF samples as obtained contain ~ 1% lactic acid and ~ 20 μM H_2_O_2 _[9,29].

To avoid conflating endogenous vaginal bacteria with the cultured bacteria used in these experiments, bacterially-depleted VF was prepared: each collected sample was diluted with a half-volume of sterile saline (0.9% [w/v] sodium chloride), mixed thoroughly, centrifuged at 1000 *g *for three minutes, and the supernatant was drawn off for immediate use in an experiment. Pilot experiments showed that this centrifugation reduced bacterial concentrations in the diluted VF by a factor of approximately 10^6^, from a pre-centrifugation mean of 5.6 × 10^7 ^cfu/mL to a post centrifugation mean of 4.0 × 10^1 ^cfu/mL (data not shown). Rather than pooling VF samples for use in experiments, individual samples from at least four different participants were used in conjunction with each treatment (H_2_O_2 _or lactic acid) to assess the reproducibility of results across different VF samples.

The effect of VF on the microbicidal activities of H_2_O_2 _and lactic acid against seven prevalent species of BV-associated bacteria (*G. vaginalis*, *A. vaginae*, *P. bivia*, *P. anaerobius*, *M. curtisii*, *M. mulieris*, and *M. hominis*) and four species of vaginal lactobacilli was measured. Each organism was exposed to growth medium containing an inactivating concentration of H_2_O_2 _(3.4% w/v [1 M] with 50 mU/mL MPO at pH 7) or lactic acid (1% w/v [111 mM] at pH 4.5), with or without the addition of bacterially depleted VF to a final VF concentration of 1% or 10% (v/v). In all cases, the bacterially depleted VF was added to the experimental media and mixed for five seconds *before *the addition of bacteria. The pH of the experimental media was also checked before the addition of bacteria, and if necessary readjusted to 4.5 or 7.0. Samples were removed from control and experimental conditions after ten minutes, thirty minutes, one hour, and two hours, serially diluted, plated and enumerated as described above.

#### Statistical analysis

Results are reported as means of at least six independently repeated experiments (two replicates performed within each experiment). The difference between three or more means was tested using an ANOVA one-way analysis of variance; difference between two means was tested using a two-tailed Student's *t *test (comparisons are paired unless otherwise indicated in the results); *p *values ≤ 0.05 were considered to be statistically significant. Statistical analysis was performed using PHStat2 version 3.0 (Microsoft Excel add-on). Due to the large amount of data presented in the graphs, standard deviations have been omitted for visual clarity; however, there were no significant differences among data from different replicates or repeats of individual experiments (differences were less than a log*_10 _*unit in all cases).

## Results

The results presented here report the effects of a two-hour exposure to H_2_O_2_, lactic acid, or acetic acid, with or without the addition of VF. Data collected at shorter exposures differed only in the proportion of each bacteria inactivated, not in the relative efficacy of the anti-microbial agents, or in the effect of VF on bacterial inactivation.

### Microbicidal activity of hydrogen peroxide

**Figure 1:**
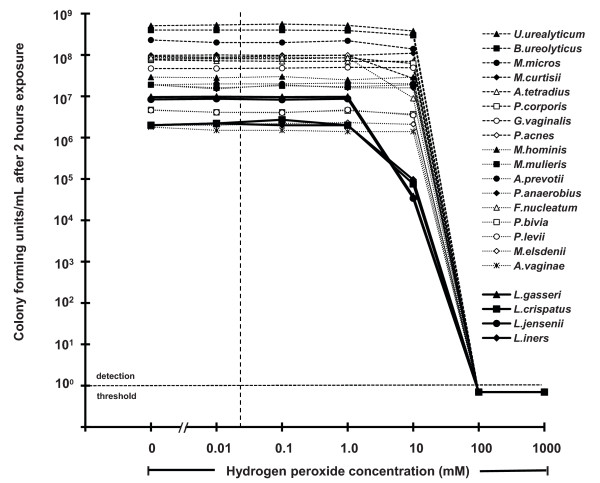
**Microbicidal activity of hydrogen peroxide (H_2_O_2_) with 50 mU/mL human myeloperoxidase (MPO) at pH 7, against four species of vaginal lactobacilli (solid lines) and seventeen species of bacteria associated with bacterial vaginosis (BV) (broken lines)**. The vertical dashed line indicates the concentration of H_2_O_2 _measured in vaginal fluid (VF) from women with a lactobacilli-dominated microbiota (~ 23 μM).

**Figure 2:**
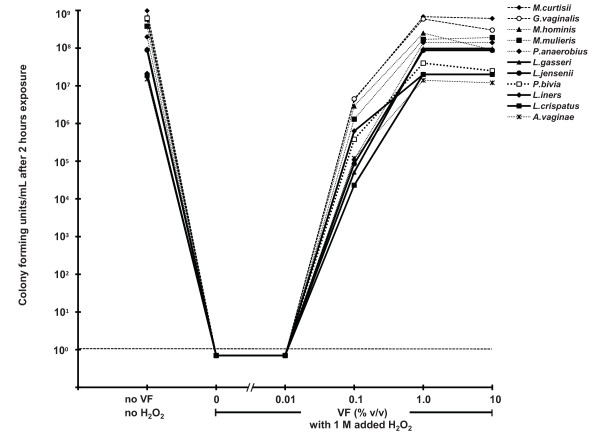
**The blocking effect of bacterially-depleted VF on the microbicidal activity of 1 M H_2_O_2 _with 50 mU/mL MPO, which is otherwise sufficient to inactivate completely four species of lactobacilli (solid lines) and seven species of BV-associated bacteria (broken lines)**.

A two-hour anaerobic exposure to 100 mM H_2_O_2 _with 50 mU/mL MPO at pH 7 reduced the viability of all four vaginal lactobacilli species and all seventeen BV-associated bacterial species to undetectable levels (a measured reduction of between 10^6 ^and 10^9 ^organisms per mL, depending on the initial bacterial concentration). This demonstrates the broad-spectrum activity of H_2_O_2 _(Figure 1). However, this concentration is approximately 50-fold higher than lactobacilli are capable of producing even under optimal aerobic, low-antioxidant conditions, and approximately 5,000-fold higher than the estimated H_2_O_2 _concentration *in vivo *(VF). The microbicidal activity of H_2_O_2 _was not enhanced by lower pH; indeed, at pH 4.5 H_2_O_2 _with MPO produced less reduction in viability than at pH 7, presumably due to reduced activity of MPO at the lower pH (data not shown). The addition of just 1% bacterially depleted VF completely blocked the microbicidal activity of 1 M H_2_O_2 _with 50 mU/mL myeloperoxidase at pH 7; no significant inactivation was detected in any of the eleven bacterial species tested (Figure 2). It is worth emphasizing that we tested the effect of 1M H_2_O_2 _to determine the potency of VF for blocking the microbicidal activity of H_2_O_2_, but this concentration is higher than is physiologically plausible_._

### Microbicidal activity of acidity, lactic acid, and acetic acid

**Figure 3:**
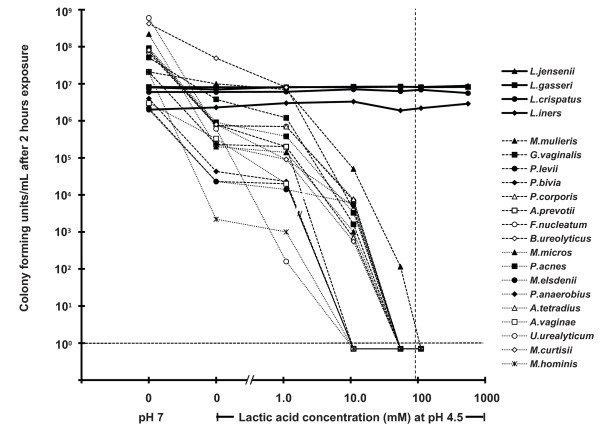
**Microbicidal activity of lactic acid at pH 4.5 against four species of vaginal lactobacilli (solid lines) and seventeen species of BV-associated bacteria (broken lines)**. The vertical dashed line indicates the mean concentration of lactic acid measured in VF from women with a lactobacilli-dominated vaginal microbiota (93 mM).

**Figure 4:**
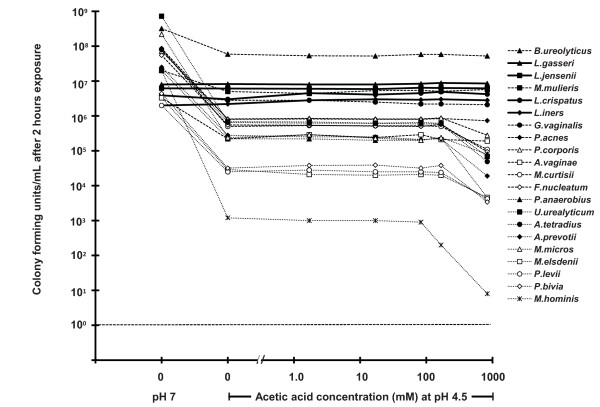
**Microbicidal activity of acetic acid at pH 4.5 against four species of vaginal lactobacilli (solid lines) and seventeen species of BV-associated bacteria (broken lines)**.

Acidity alone (pH 4.5 compared to pH 7) reduced the viability of all seventeen BV-associated bacteria (a reduction of between two-fold and 10^4^-fold, depending on bacterial species) after two hours exposure, but had no effect on any of the four lactobacilli species tested ("0, pH 7", and "0, pH 4.5" data points in Figure 3). Similarly, acetic acid caused essentially no additional inactivation compared to pH 4.5 alone except at 0.8 M (5% w/v), where it caused only partial additional inactivation (Figure 4).

**Figure 5:**
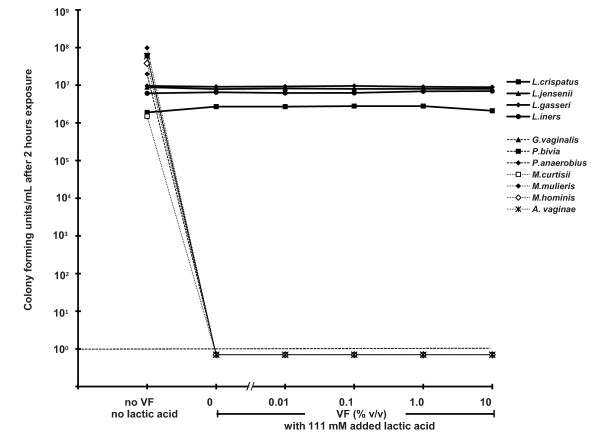
**The effect of bacterially-depleted VF on the microbicidal activity of 100 mM lactic acid at pH 4.5, which is otherwise sufficient to inactivate completely seven species of BV-associated bacteria (broken lines), but not four species of lactobacilli (solid lines)**.

In striking contrast, the addition of lactic acid greatly increased the microbicidal potency at pH 4.5: 0.5% w/v (56 mM) lactic acid, a concentration and acidity at the lower end of the range observed in a healthy vaginal environment [29], dramatically reduced the viability of all BV-associated species, with all but one (*M. mulieris*) reduced to undetectable levels (a measured reduction of 10^6^-fold to 10^8^-fold depending on the initial bacterial concentration). Not surprisingly given that they produce lactic acid, all four lactobacilli species tested were unaffected by lactic acid at a concentration of 0.5% w/v (56 mM); indeed, the lactobacilli were unaffected by 10% w/v (1110 mM) lactic acid, an order of magnitude more lactic acid than we measured in VF from women with a lactobacilli-dominated vaginal microbiota [[29], and manuscript in preparation]. Moreover, addition of 1% or 10% v/v bacterially-depleted VF did not change the microbicidal effect of 56 mM lactic acid at pH 4.5; all BV-associated bacteria tested were completely inactivated, and lactobacilli were unaffected (Figure 5). The microbicidal activity of lactic acid required low pH; at pH 7.0 lactic acid did not inactivate any of the bacteria tested (data not shown).

## Discussion

Here we report that physiologically plausible concentrations of H_2_O_2 _had no microbicidal activity, while a supraphysiologic concentration of exogenous H_2_O_s _(0.34% w/v, 100 mM) just high enough to inactivate BV-associated bacteria more potently inactivated vaginal lactobacilli. In contrast, physiological concentrations of lactic acid (0.5% w/v, 56 mM) at pH 4.5 completely inactivated sixteen of the seventeen species of BV-associated bacteria tested; pH 4.5 is the highest pH likely to occur when lactobacilli dominate the vaginal bacterial community [[25], and manuscript in preparation]. The differential effect observed was opposite to that expected if lactobacilli suppress BV-associated bacteria with H_2_O_2_. This also argues against the possibility that H_2_O_2 _might be protective at high local concentrations. Additionally, as we have previously reported, addition of only 1% VF blocks the microbicidal activity of H_2_O_2_-producing strains of lactobacilli even in an optimized, aerobic, low-antioxidant buffer system [4].

At low pH, small weak acids like acetic acid and lactic acid become uncharged free acids that are lipid soluble, membrane permeant, and capable of acidifying the cytosol. The p*K_a _*for acetic acid is ~ 4.8, thus when vaginal pH is < 4, acetic acid exists primarily as the uncharged free acid. In contrast, the p*Ka *for lactic acid is ~ 3.8 and thus at vaginal pH much of it is the far less membrane permeant lactate anion. Acetic acid is both smaller and more lipid soluble than lactic acid and hence acetic acid is expected to acidify the cytosol more rapidly than lactic acid and be more rapidly bactericidal than lactic acid. Despite this expectation, we found that acetic acid had no detectable effects until its concentration was increased to 5% (household vinegar). Therefore, the marked inhibition of BV-associated bacteria by lactic acid clearly indicates that the antimicrobial action of lactic acid is not based simply on cytosolic acidification. Instead, as suggested by other studies, lactic acid appears to have specific effects, for example, disturbing the cell membranes of Gram-negative bacteria [22].

Menstrual fluid neutralizes the vagina, and we found that at pH 7 lactic acid had no microbicidal activity against BV-associated bacteria. This result is consistent with the clinical observation of BV recurrence after menses [30,31].

Our observations carry the caveat of all *in vitro *observations, namely that the activities of H_2_O_2_, lactic acid, and acetic acid *in vitro *may not be the same as *in vivo*.

Additionally, inactivation of lactobacilli during a transient anaerobic exposure to exogenous H_2_O_2 _may not reveal tolerance mechanisms that might occur in aerobic conditions when lactobacilli can produce H_2_O_2_. However, lactobacilli inactivate themselves by endogenous H_2_O_2 _production [32,33], indicating that they do not possess adequate mechanisms to overcome H_2_O_2 _toxicity. Lactobacilli and BV-associated bacteria were used at relatively high concentrations for these experiments; however, the concentrations used here reflect those found *in vivo*. Finally, we investigated single species *in vitro*, not combinations, and there may be synergistic effects of combinations of BV-associated bacteria that might significantly alter the results *in vivo*.

## Conclusions

We found that addition of hydrogen peroxide was not microbicidal at physiologically plausible concentrations. When supplied at microbicidal concentrations, H_2_O_2 _inactivated vaginal lactobacilli somewhat more potently than BV-associated bacteria. Conversely, addition of lactic acid at physiological concentrations *was *microbicidal against BV-associated bacteria, but had no effect on vaginal lactobacilli. Additionally, the presence of VF blocked the microbicidal activity of H_2_O_2 _but not of lactic acid. We conclude that H_2_O_2 _production by lactobacilli is an implausible mechanism for suppressing BV-associated bacteria *in vivo*, and that lactic acid production at rates that acidify the vagina may potently suppress BV-associated bacteria.

## Competing interests

Based on the results reported in this paper, the authors have applied for patents on devices and methods for sustained release of lactic acid, with assignment to ReProtect and Johns Hopkins University. TRM and RAC own equity in ReProtect.

## Authors' contributions

DEOH designed the study, collected the data, analyzed the data, and prepared the manuscript. TM and RC participated in data analysis and the preparation of the manuscript. All of the authors read and approved the final manuscript.

## Pre-publication history

The pre-publication history for this paper can be accessed here:

http://www.biomedcentral.com/1471-2334/11/200/prepub
